# Percutaneous Fibrin Gel Injection under C-Arm Fluoroscopy Guidance: A New Minimally Invasive Choice for Symptomatic Sacral Perineural Cysts

**DOI:** 10.1371/journal.pone.0118254

**Published:** 2015-02-23

**Authors:** Wei Jiang, QuanHe Qiu, Jie Hao, XiaoJun Zhang, Wei Shui, ZhenMing Hu

**Affiliations:** Department of Orthopaedic Surgery, The First Affiliated Hospital of Chongqing Medical University, Chongqing, China; University of Michigan, UNITED STATES

## Abstract

**Background:**

Symptomatic sacral perineural cysts are a common cause of chronic pain. Surgery is one choice for symptom relief but has a high risk of cyst recurrence and complications. As a simple and safe method to manage symptomatic sacral perineural cysts, C-arm fluoroscopy-guided fibrin gel injection may represent a new minimally invasive alternative. To evaluate the efficacy of this new method, we conducted a retrospective study of 42 patients.

**Methods and Findings:**

From June 2009 to August 2012, a total of 42 patients with symptomatic sacral perineural cysts underwent C-arm fluoroscopy-guided percutaneous fibrin gel injection therapy. Patient outcomes in terms of improvements in pain and neurologic function were evaluated during a follow-up period of 13–39 months. The preoperative and postoperative pain severity were assessed according to a 10-cm visual analog pain scale, and imaging changes were evaluated by magnetic resonance imaging. We also assessed postoperative complications. Most patients experienced benefit from the procedure: twenty-five patients (59.5%) reported excellent recovery, eleven (26.2%) reported good recovery, three (7.1%) reported fair recovery, and three (7.1%) reported poor recovery. The overall effectiveness rate (excellent and good recoveries) was 85.7%. No serious postoperative complications were observed.

**Conclusion:**

Percutaneous fibrin gel injection under C-arm fluoroscopy guidance could be a simple, safe and effective treatment option for symptomatic sacral perineural cysts.

## Introduction

Sacral perineural cysts, which are also referred to as Tarlov cysts, were first described by Tarlov in 1938 as an incidental finding at autopsy [1]. The cysts are collections of cerebrospinal fluid (CSF) involving the extradural components of sacral or coccygeal nerve roots [2] that have been overlooked clinically because the cysts often occur asymptomatically. In a series of 500 consecutive magnetic resonance imaging (MRI) scans of the lumbosacral spine, Paulsen et al. [3] reported that the incidence of symptomatic cysts was approximately 1%. If the sacral perineural cysts become large enough to compress nerve roots or the sacral nerve plexus, then they may cause symptoms including lower back pain, sacrococcygeal pain, perineal pain, sacral nerve root pain (sciatic pain), leg weakness, neurogenic claudication, bowel and bladder dysfunction, and even sexual dysfunction.

There has been no consensus with regard to the optimal treatment of symptomatic sacral perineural cysts since the condition was first described by Tarlov [4]. Steroid medication, analgesics, non-steroidal anti-inflammatory medication and physical therapy have been used as conservative managements to treat these symptomatic lesions, with variable results [5], [6]. Surgical decompression can be a complicated operation, with the risk of unsatisfactory results due to nerve injury, hemorrhage, CSF leaks, or recurrence [3], [7]. The effects of lumboperitoneal shunts, subarachnoid cyst shunts and lumbar CSF drainage have been unsatisfactory for symptomatic sacral perineural cysts [8–10]. Recently, Patel et al. [11] adopted computed tomography (CT) fluoroscopy-guided percutaneous fibrin gel injection to treat symptomatic sacral perineural cysts presenting with back pain. We know that compared with CT fluoroscopy, C-arm fluoroscopy is more economical and a more commonly used intraoperative guidance tool. Our retrospective study was designed to evaluate a new method, percutaneous fibrin gel injection guided by C-arm fluoroscopy rather than by CT fluoroscopy. From June 2009 to August 2012, 42 patients with symptomatic sacral perineural cysts underwent C-arm fluoroscopy-guided percutaneous fibrin gel injection therapy in our hospital. We assessed whether the injection of fibrin gel into a perineural cyst could be successfully completed under C-arm fluoroscopy guidance and whether this new minimally invasive treatment was safe and effective for symptomatic sacral perineural cysts.

## Materials and Methods

### Patient Population

Between June 2009 and August 2012, a total of 49 patients with symptomatic sacral perineural cysts were treated in our hospital. Ultimately, 42 patients (20 men, 22 women; mean age, 34.3 years; range, 22–56 years; mean disease duration, 20.8 months; range, 7–59 months) who met the inclusion/exclusion criteria were enrolled in the study. The inclusion criteria were as follows: 1) sacral perineural cysts confirmed by MRI; 2) symptoms such as leg pain and numbness, muscle atrophy, lumbosacral pain, perineum/saddle discomfort (pain and numbness), or bowel and bladder dysfunction; 3) experiencing symptoms for more than 6 months and failing conservative treatments (medical treatments with anti-inflammatory drugs, neurotrophic drugs, and physical therapy) for more than 3 months; and 4) no history of trauma or infection. The exclusion criteria were anorectal, gynecological, urological, or lumbar vertebral diseases, including lumbar disc herniation and lumbar spondylolisthesis.

Of the 42 patients, 5 patients had pain in the legs with or without muscle atrophy, and 33 patients experienced lumbosacral pain accompanied by numbness of the perineal region or lateral posterior aspect of the legs (one 37-year-old male patient suffered from pain at the root of the penis area that prevented him from having intercourse). Four patients exhibited symptoms of cauda equina compression with sensory abnormalities in the perineum/saddle area, bladder and bowel dysfunction, or a burning sensation around the anus (Table 1). Twenty patients reported symptoms that were associated with postural changes; standing or walking worsened the pain and numbness, while bed rest significantly alleviated these symptoms.

**Table 1:** Summary of cyst characteristics and clinical symptoms in 42 patients.

Cyst Location	No. of Patients	Clinical Symptoms
L5-S1	5	Leg pain, muscle atrophy, numbness in saddle region and legs
S1-S2	21*	Lumbosacral pain, bladder and bowel dysfunction, and saddle discomfort (numbness)
S2-S3	17*	Lumbosacral pain, bladder and bowel dysfunction, and burning sensation around the anus

* One patient had multiple cysts that occupied 2 segments (S1-S2 and S2-S3).

All patients underwent plain radiographs and 1.5T MRI prior to admission. The spinal levels of the cysts were as follows: L5-S1 (n = 5), S1–2 (n = 21) and S2–3 (n = 17). Of the 42 patients, 31 had a single cyst while the remaining 11 patients had multiple cysts.

### Ethics Statement

This study was approved by the Institutional Review Board of the First Affiliated Hospital of Chongqing Medical University, and all aspects of the study complied with the Declaration of Helsinki. The Institutional Review Board of the First Affiliated Hospital of Chongqing Medical University also waived the requirement for patient consent because this study was retrospective, the data were analyzed anonymously and patient care was not affected by the study.

### Surgical Procedures

**Fig 1:**
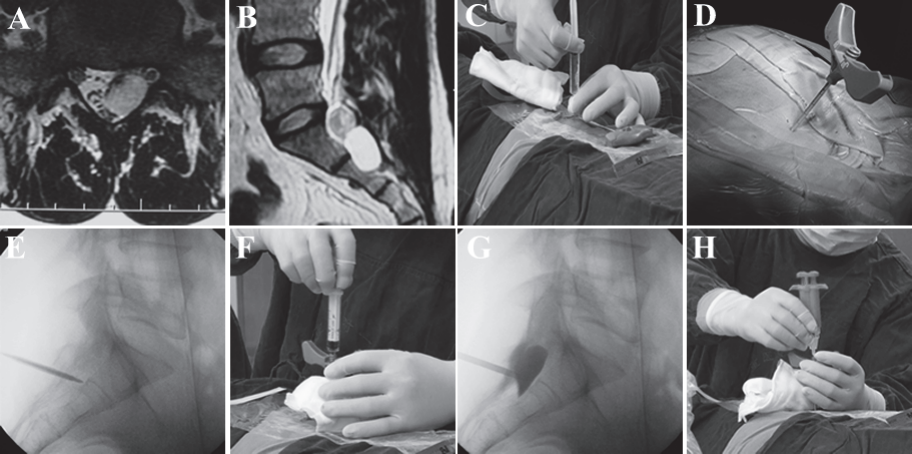
Procedure of fibrin gel therapy under C arm fluoroscopy machine guidance for symptomatic sacral perineural cysts. A and B. Transverse and sagittal T2WI MRI (prior to admission) show the location of cysts. C. Local anesthesia is induced with 2% lidocaine from skin to periosteum of sacral canal after needle insertion point is confirmed. D and E. Bone-puncture needle is used to puncture the sacral lamina and is inserted into the middle of the cyst guided by the C arm x-ray machine. F and G. CSF is aspirated and iohexol is injected to confirm the location of the bone-puncture needle in the cyst. H. Fibrin gel is injected into the cyst.

An iodine allergy test was necessary for all patients prior to the procedure. Patients were placed in the prone position on the C-arm table for the entire procedure. The cysts were first localized based on the MRI images obtained at hospital admission; localization was based on axial (Fig. 1A) and sagittal (Fig. 1B) views, with the sacral vertebra selected as the reference point. During the procedure, a cross-shaped Kirschner wire was placed on the skin of the sacral region, and the image at the intersection point under fluoroscopy was used to locate the cysts again. Finally, the middle area of the cyst (the image at the intersection point of the cross-shaped Kirschner wire in the middle area of the cyst’s MRI image) was selected as the needle insertion point. The skin was prepped and draped in the routine fashion. After local anesthesia with 2% lidocaine from the skin to the periosteum of the sacral canal (Fig. 1C), an 11-gauge bone-puncture needle (GuanLong Corporation, ShanDong, China) was used to puncture through the sacral lamina and inserted into the middle of the cyst under C-arm fluoroscopy guidance (Fig. 1D). The intraoperative C-arm fluoroscopy image showed a metal needle shadow within the cavity (Fig. 1E), confirming that the puncture needle was inserted into the cyst. The CSF was aspirated slowly to prevent any discomfort caused by sudden decompression. Aspiration was continued until no more CSF could be removed, and the final volume was recorded (mean 3 ml; range, 2–5 ml) (Fig. 1F). An iohexol injection (1–2 ml, Yangtze River Pharmaceutical Group Co., LTD, China) was injected into the cyst to again confirm that the puncture needle was placed in the cyst (Fig. 1G). Next, the iohexol injection was slowly and completely aspirated. After drainage of the cyst, fibrin gel was injected into the cavity of the cyst. The fibrin gel was used according to the manufacturer’s instructions (Pu Ji Medical Technical Development Limited Company, China). Fibrin gel is composed of a main gel (containing fibrinogen dissolved in a phosphate buffer solution) and a thrombin catalyst (dissolved in a calcium chloride solution). The main gel and thrombin catalyst were dissolved in a specific deliquescent substance (provided by the manufacturer) and loaded into 2 syringes that were connected via a connecting stent. Equal volumes of the main gel and thrombin catalyst solutions were injected into the cyst cavity simultaneously until the total volume was equal to the amount of CSF aspirated from the cyst (Fig. 1H). The injection was stopped when pain occurred in the lumbosacral region or innervation area. For the patients with multiple cysts, only the 1 or 2 cysts that were considered to be the chief culprit(s) of the symptoms underwent fibrin gel injection.

### Postoperative Management

Patients without any postoperative discomfort were advised to spend at least 1 day on bed rest. An average of 3 days (range, 2–4 days) of bed rest was necessary for the patients who felt pain at the puncture site after the procedure. For the patients who experienced side effects from the fibrin gel therapy (such as headache, low grade fevers, nausea and vomiting), two days of 20% mannitol 250 ml and dexamethasone 10 mg and one day of prophylactic antibiotics were administered in addition to an average of 3 days (range, 2–4 days) of bed rest.

### Evaluation of Clinical Outcomes

The effect of the operation was assessed by the pain index, functional improvement and imaging findings before and after the fibrin gel therapy. The preoperative and postoperative pain severity was evaluated according to a 10-cm visual analog pain scale (VAS). The evaluation criteria cited from Zhang et al. [12] were as follows: (1) excellent: all symptoms and signs disappeared, and the patient returned to his or her regular employment; (2) good: symptoms and signs in the legs and perineal region disappeared, but the pain in the lumbosacral region persisted, though it did not disturb the patient’s regular work; (3) fair: no improvement in clinical symptoms, but a shrink in cyst size on the imaging study; (4) poor: either no improvement in clinical symptoms and no observed changes in cyst size in imaging studies or recurrence.

### Statistical Analysis

Comparisons between preoperative and postoperative outcomes were made by a paired-samples *t* test (Statistical Package for the Social Sciences, Version 17.0; SPSS, Chicago, IL, USA), and p<0.05 was considered statistically significant.

## Results

Fibrin gel injection was successfully completed under C-arm fluoroscopy guidance for all the cases. All patients were observed for 13–39 months (an average follow-up of 24 months). Most patients significantly benefited from the operation (Table 2). Twenty-five patients (59.5%), whose recovery was evaluated as excellent returned to their regular employment without any symptoms and signs. Eleven patients who experienced partial resolution of their discomfort (symptoms and signs in the legs and perineal region disappeared; the pain in the lumbosacral region persisted but did not interfere with the patient’s regular work) were deemed to have good recovery (26.2%). Three patients without improvements in pain or function but with a shrinkage of the cyst size were rated as fair recovery (7.1%). Three patients who had no improvement in their clinical symptoms and no observed changes in cyst size in imaging studies were rated as poor recovery (7.1%). The overall effective rate (excellent and good recoveries) was 85.7%, and no recurrence was observed.

**Table 2:** Recovery from pain after a mean follow-up of 24 months in 42 patients*.

No. of Patients					
Intervention	Painless	Mild Pain	Moderate Pain	Severe Pain	Total
Preoperative	4	2	21	15	42
Postoperative	25	11	3	3	42

*Recovery was assessed by the VAS (VAS of 0, painless; 1–3, mild pain; 4–6, moderate pain; and 7–10, severe pain). Paired-samples *t* test showed a significant difference between the preoperative and postoperative periods (P＜0.05), which suggested that the fibrin gel therapy could significantly improve the symptoms of pain.

No postoperative infection, nerve damage, or CSF leaks occurred in any of the patients. Sanguineous fluid was aspirated during the procedure in 6 cases, but no postoperative complications were observed in these patients. Seven patients reported headache, low grade fever (37.5°C-37.9°C), or nausea and vomiting without neck stiffness, which are known possible side effects of fibrin gel injection therapy. The post-procedure discomfort disappeared completely after a treatment of two days of 20% mannitol 250 ml and dexamethasone 10 mg and one day of prophylactic antibiotics in addition to an average of 3 days (range, 2–4 days) of bed rest.

**Fig 2:**
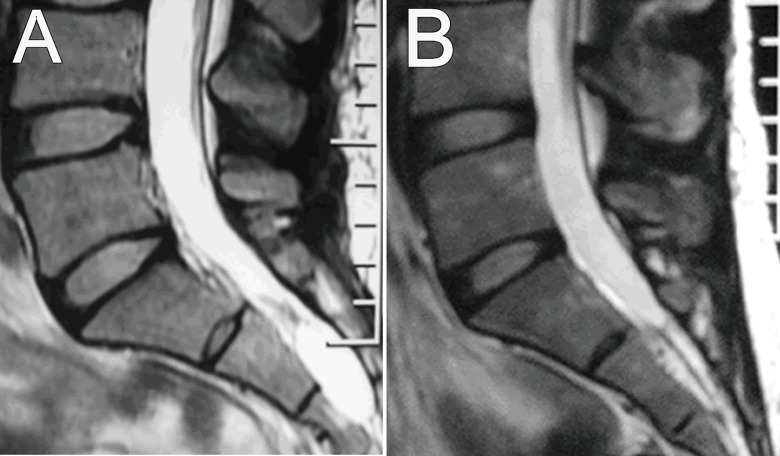
MRI study of cyst disappearance. A. Sagittal T2WI MRI shows cysts before fibrin gel therapy. B. Sagittal T2WI MRI obtained 24 months after operation shows that the cysts disappeared completely.

**Fig 3:**
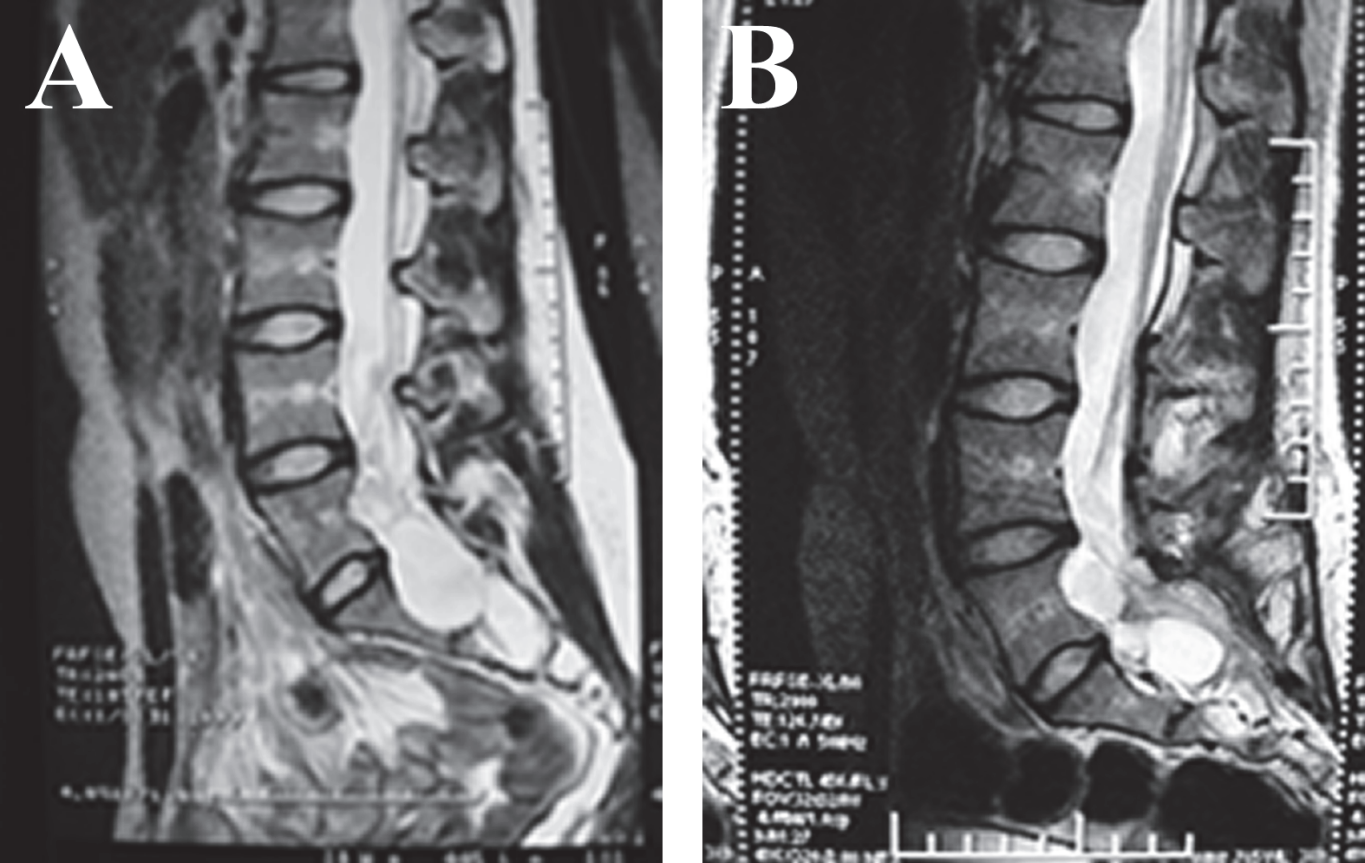
MRI study of shrinkage of the cyst. A. Sagittal T2WI MRI shows multiple cysts before fibrin gel therapy. B. Sagittal T2WI MRI obtained 18 months after operation shows that the cysts became smaller but did not disappear completely.

**Fig 4:**
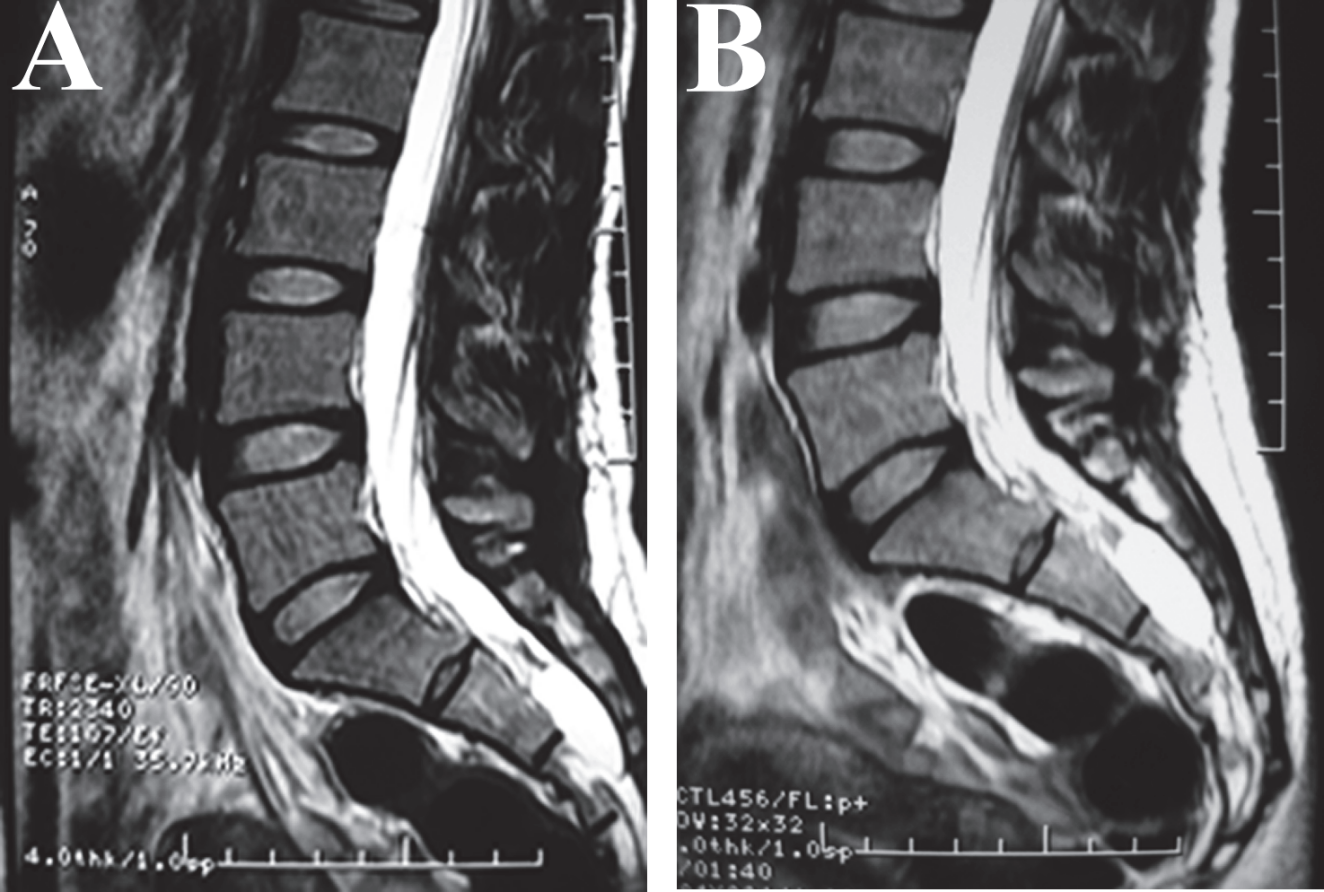
MRI study of no change of the cyst. A. Sagittal T2WI MRI shows a single cyst located in S2-S3 before fibrin gel therapy. B. Sagittal T2WI MRI obtained 10 months after operation shows that the size of the cyst did not change.

We also estimated the changes in the imaging findings by MRI during the follow-up period after fibrin gel injection therapy; the time points were 3 months, 6 months, 9 months, 12 months, 18 months, 24 months, 30 months, 36 months and 42 months after therapy (in some cases the MRI reexamination was not performed punctually). The results indicated that 25 cysts disappeared completely (Fig. 2) and 14 cysts significantly decreased in size (Fig. 3); the size of these cysts did not increase during the follow-up interval. We also observed 3 cysts that had no change in size during the follow-up (Fig. 4).

## Discussion

Sacral perineural cysts are defined as CSF-filled saccular lesions located in the extradural space of the sacral spinal canal. They are formed within the nerve root sheath at the dorsal root ganglion. Sacral perineural cysts were first described by Tarlov in 1938; different types of spinal cysts were subsequently described by others [13]. Nabors et al. [14] classified the spinal cysts into the following 3 types: Type I (extradural meningeal cysts without spinal nerve root fibers); Type II (extradural meningeal cysts with spinal nerve root fibers, Tarlov cysts); and Type III (spinal intradural meningeal cysts). To date, no consensus has been reached regarding the etiology of sacral perineural cysts. The most important hypotheses have included the following: (1) inflammation of nerve root cysts followed by inoculation of fluid and arachnoid proliferation along and around the sacral nerve root; (2) breakage of venous drainage in the perineurium and epineurium secondary to hemosiderin deposition after trauma; and (3) congenital origin [8], [15], [16]. A genetic origin or degeneration are also considered to be important factors involved in the pathogenesis of sacral perineural cysts [9], [17], [18]. Most researchers consider the cysts to be congenital defects. The cysts are created from dilated sheaths that have micro-connections to the subarachnoid space known as “ball-valve” communications (also called “traffic pores”), through which the CSF can flow into the sheath but cannot exit from it. The cysts enlarge progressively because of the continuous single infusion of the CSF [19], [20].

Compared with myelography (an invasive imaging modality that can detect communication of the sacral perineural cysts with the subarachnoid space), MRI is noninvasive and considered to be the gold-standard modality to detect sacral perineural cysts. MRI is also used to study the relationship between the cysts and the surrounding structures and to plan surgical treatment [13]. With MRI, the cyst appears as low signal on T1WI and high signal on T2WI. In our study, we used MRI to detect the cysts and to locate them during the procedure. (Fig. 1A, 1B, 1E).

Although most sacral perineural cysts are asymptomatic and the incidence of symptomatic cysts was approximately 1% or less in some reports [3], [7], [17], this does not mean that the problem should be overlooked. If sacral perineural cysts become large enough to compress nerve roots or the sacral nerve plexus, then they may cause obvious discomfort such as lower back pain, sacrococcygeal pain, perineal pain, sacral nerve root pain (sciatic pain), leg weakness, neurogenic claudication, bowel and bladder dysfunction, and even sexual dysfunction [15], [18], [21]. In our study, over 80% (35/42) of patients complained that the discomfort caused by sacral perineural cysts seriously disturbed their daily lives. Pain (such as lumbosacral pain and leg pain) is the most important symptom of symptomatic sacral perineural cysts, and approximately 90% patients in our study sought treatment due to pain. In our study, the VAS was used to assess the pain severity before and after operation.

Thus far, various methods have been used to treat symptomatic sacral perineural cysts. Conservative treatments include medical (with analgesic and nonsteroidal anti-inflammatory medications) and physical therapy [6]. Surgical methods include two options: a direct microsurgical approach [7], [8], [15], [22] (cyst resection with or without muscle patch, cyst fenestration and imbrication, cyst resection with neck ligation and cyst resection combined with excision of the related nerve root); and diversion of CSF flow (percutaneous cyst drainage with or without fibrin gel injection, lumboperitoneal shunt, or cystosubarachnoid shunt) [3], [10], [11], [12]. Although some researchers recommend the direct microsurgical approach for selected symptomatic patients and have reported good results, surgeons should be vigilant regarding the main drawbacks of surgical management, which include a high risk of recurrence and complications (such as nerve damage, meningitis and CSF leakage). The lumboperitoneal shunt procedure has produced uncertain results and carries the accompanying risk of infection. Symptoms often recur after percutaneous cyst drainage due to recollection of the CSF. Recently, a new method of CT-guided percutaneous injection of fibrin gel after cyst drainage has been reported to treat symptomatic sacral perineural cysts and has achieved 86.8% positive outcomes [23]. In our study, we treated 42 cases of symptomatic sacral perineural cysts by fibrin gel injection under C-arm fluoroscopy guidance and obtained comparative results. In the follow-up estimates, 85.7% of patients experienced positive outcomes after fibrin gel injection, and most patients experienced complete resolution of their clinical symptoms and returned to their regular employment. Moreover, none of our patients had complications such as nerve damage, meningitis or CSF leak. Compared with CT fluoroscopy, C-arm fluoroscopy is more economical and is common as a tool for intraoperative guidance. Our results indicated that the fibrin gel injection procedure could be completed successfully under C-arm fluoroscopy guidance. We believe that more patients and doctors might be able to benefit from this new method.

As reported, most cysts disappear completely several months after fibrin gel therapy, and these results may be attributed to the characteristics of the fibrin gel itself [11], [12], [23], [24]. The cyst cavities are filled and the traffic pores between the cysts and the subarachnoid space are sealed by fibrin gel. Subsequently, the cysts are absorbed, resulting in fibrin gel resorption by fibroblast proliferation and fibrous scar formation. We also observed similar results in our study, and the follow-up MRI images indicated that 36 cysts completely disappeared or shrank to different degrees after the fibrin gel injection therapy.

Although often asymptomatic, sacral perineural cysts should not be overlooked because enlarged sacral perineural cysts can result in various and sometimes serious discomforts. All neurosurgeons and orthopedic spine surgeons should pay close attention to symptomatic sacral perineural cysts. Conservative treatment with anti-inflammatory drugs, neurotrophic drugs, and physical therapy should be offered initially for patients with mild pain or sensory abnormalities. Fibrin gel therapy could be a good choice for patients who fail conservative treatment or experience severe pain and nerve dysfunction. We adopted the new method of percutaneous fibrin gel injection under C-arm fluoroscopy guidance to treat symptomatic sacral perineural cysts and observed good clinical results. Of course, the results should be interpreted with caution because of the relatively small number of patients studied and the limited duration of follow-up in our study.

## Conclusions

Sacral perineural cysts are worthy of research due to their potential to cause patients serious discomfort. In our study, a new minimally invasive method, C-arm fluoroscopy-guided percutaneous fibrin gel injection, was used to manage symptomatic sacral perineural cysts and produced satisfactory effects. We recommend that C-arm fluoroscopy-guided percutaneous fibrin gel injection may be a simple, safe, and effective therapy choice for symptomatic sacral perineural cysts.

## Supporting Information

S1 Dataset Raw data of 42 patients. (RAR)
